# Supplementary material for: Vaccine-Acquired SARS-CoV-2 Immunity versus Infection-Acquired Immunity: A Comparison of Three COVID-19 Vaccines
Source: Vaccines (Basel). 2022 Dec 15;10(12):2152. doi: 10.3390/vaccines10122152 (PMC9782527; doi:10.3390/vaccines10122152)
Supplement: Supplementary file 1 [file vaccines-10-02152-s001.zip › vaccines-2070874-supplementary.pdf]

**Table S1.** Clinical information of vaccinated participants.

| Group | Participant | Age at visit (years) | Days Post-onset or Days post last vaccination dose | Gender | Race | Ethnicity | Notable Comorbidities | Confirmation of COVID test |
|---|---|---|---|---|---|---|---|---|
| J&J | J&J-01 | 33.1 | 92 | F | White | Non-hispanic | None | n/a |
| | J&J-02 | 23.2 | 115 | F | White | Non-hispanic | None | n/a |
| | J&J-03 | 36.5 | 94 | F | White | Non-hispanic | None | n/a |
| | J&J-04 | 42.7 | 103 | F | White | Non-hispanic | Bipolar | n/a |
| | J&J-05 | 30.0 | 105 | M | White | Non-hispanic | None | n/a |
| | J&J-06 | 42.3 | 118 | F | Asian | Non-hispanic | Uterine fibroid | n/a |
| | J&J-07 | 51.8 | 117 | F | Black | Non-hispanic | Asthma | n/a |
| | J&J-08 | 35.2 | 119 | M | White | Non-hispanic | Asthma, GERD | n/a |
| | J&J-09 | 59.2 | 119 | F | White | Non-hispanic | None | n/a |
| | J&J-10 | 39.6 | 138 | F | other | Non-hispanic | prediabetes, migranes, HPV infection | n/a |
| | J&J-11 | 32.2 | 120 | M | White | Non-hispanic | None | n/a |
| | J&J-12 | 49.2 | 100 | M | White | Non-hispanic | asthma, hyperlipidemia, WPW syndrome | n/a |
| | J&J-13 | 36.2 | 141 | F | White | Non-hispanic | None | n/a |
| | J&J-14 | 39.5 | 140 | F | White | Non-hispanic | mononucleosis | n/a |
| | J&J-15 | 48.8 | 134 | F | White | Non-hispanic | None | n/a |
| | J&J-16 | 35.6 | 85 | F | White | Non-hispanic | None | n/a |
| | J&J-17 | 23.6 | 141 | F | White | Non-hispanic | None | n/a |
| | J&J-18 | 33.9 | 97 | F | White | Non-hispanic | None | n/a |
| | J&J-19 | 28.2 | 119 | F | other | Non-hispanic | None | n/a |
| | J&J-20 | 75.9 | 71 | F | White | Non-hispanic | None | n/a |
| Pfizer | Pfizer-01 | 25.0 | 107 | M | Asian | Non-hispanic | None | n/a |
| | Pfizer-02 | 24.7 | 108 | F | White | Non-hispanic | None | n/a |
| | Pfizer-03 | 33.3 | 109 | F | Asian | Non-hispanic | None | n/a |

| | | | | | | | |
|---|---|---|---|---|---|---|---|
| Pfizer-04 | 45.1 | 138 | F | White | Non-hispanic | migraine, uterine cyst | n/a |
| Pfizer-05 | 41.9 | 118 | M | Asian | Non-hispanic | None | n/a |
| Pfizer-06 | 30.4 | 118 | F | Asian | Non-hispanic | None | n/a |
| Pfizer-07 | 40.4 | 118 | F | Asian | Non-hispanic | None | n/a |
| Pfizer-08 | 53.4 | 116 | M | Black | Non-hispanic | None | n/a |
| Pfizer-09 | 37.5 | 118 | F | White | Non-hispanic | None | n/a |
| Pfizer-10 | 36.8 | 110 | M | Asian | Non-hispanic | None | n/a |
| Pfizer-11 | 42.6 | 81 | F | White | Non-hispanic | Asthma, GERD, hyperlipidemia, HPV | n/a |
| Pfizer-12 | 38.4 | 134 | F | Asian | Non-hispanic | None | n/a |
| Pfizer-13 | 32.4 | 121 | F | Asian | Non-hispanic | PCOS | n/a |
| Pfizer-14 | 54.6 | 132 | F | Other | Non-hispanic | Arthritis, Hashimito's disease, psoriasis | n/a |
| Pfizer-15 | 30.4 | 124 | M | Asian | Non-hispanic | None | n/a |
| Pfizer-16 | 28.5 | 123 | F | Asian | Non-hispanic | Hepatitis B, hyperhidrosis, ovarian cyst, psoriasis | n/a |
| Pfizer-17 | 36.4 | 132 | F | White | Non-hispanic | HPV, hypothyroidism, thyroid cancer | n/a |
| Pfizer-18 | 30.6 | 125 | F | Asian | Non-hispanic | None | n/a |
| Pfizer-19 | 56.9 | 127 | M | White | Non-hispanic | None | n/a |
| Pfizer-20 | 43.4 | 133 | F | White | Non-hispanic | Morbid obesity, PCOS, pancreatitis, OSA | n/a |
| Pfizer-21 | 26.9 | 101 | F | Black | Hispanic | Asthma, ovarian cyst | n/a |
| Pfizer-22 | 46.2 | 135 | F | White | Non-hispanic | Melanoma | n/a |
| Pfizer-23 | 47.0 | 134 | M | White | Non-hispanic | Tuberculosis | n/a |
| Pfizer-24 | 57.3 | 132 | M | Other | Hispanic | Diabetes | n/a |
| Pfizer-25 | 55.8 | 135 | F | Asian | Hispanic | Asthma, Arthritis, Diabetes, Cataract | n/a |
| Pfizer-26 | 31.2 | 141 | F | Asian | Hispanic | None | n/a |
| Pfizer-27 | 31.2 | 141 | F | White | Non-hispanic | Asthma | n/a |
| Pfizer-28 | 26.6 | 143 | F | White | Non-hispanic | Anxiety, depression | n/a |
| Pfizer-29 | 35.2 | 113 | F | White | Non-hispanic | ITP | n/a |
| Pfizer-30 | 62.8 | 131 | M | White | Non-hispanic | Hypertension, hyperlipidemia | n/a |
| Pfizer-31 | 39.8 | 131 | M | White | Non-hispanic | None | n/a |
| Pfizer-32 | 39.7 | 122 | F | Asian | Non-hispanic | None | n/a |
| Pfizer-33 | 37.1 | 124 | F | White | Non-hispanic | Shingles | n/a |

| | | | | | | | | |
|---|---|---|---|---|---|---|---|---|
| | Pfizer-34 | 47.0 | 129 | M | White | Non-hispanic | None | n/a |
| | Pfizer-35 | 52.3 | 138 | F | Asian | Non-hispanic | Hypertension, fibroids | n/a |
| | Pfizer-36 | 34.9 | 127 | M | Asian | Non-hispanic | Hypothyroidism | n/a |
| | Pfizer-37 | 43.4 | 127 | M | White | Non-hispanic | None | n/a |
| | Pfizer-38 | 38.9 | 113 | F | White | Non-hispanic | Anemia, asthma, ADD | n/a |
| | Pfizer-39 | 38.7 | 136 | M | Asian | Non-hispanic | None | n/a |
| | Pfizer-40 | 47.5 | 142 | M | White | Hispanic | None | n/a |
| | Pfizer-41 | 52.0 | 115 | F | White | Non-hispanic | None | n/a |
| | Pfizer-42 | 38.8 | 121 | M | Asian | Non-hispanic | None | n/a |
| | Pfizer-43 | 21.8 | 121 | F | Black | Non-hispanic | None | n/a |
| | Pfizer-44 | 49.5 | 125 | M | Asian | Non-hispanic | None | n/a |
| | Pfizer-45 | 36.1 | 123 | M | Asian | Non-hispanic | None | n/a |
| | Pfizer-46 | 34.5 | 119 | M | White | Non-hispanic | None | n/a |
| | Pfizer-47 | 29.2 | 121 | F | White | Non-hispanic | None | n/a |
| **Moderna** | Moderna-01 | 27.0 | 89 | M | White | Non-hispanic | None | n/a |
| | Moderna-02 | 53.9 | 92 | M | White | Non-hispanic | None | n/a |
| | Moderna-03 | 33.2 | 93 | F | White | Non-hispanic | GERD | n/a |
| | Moderna-04 | 30.0 | 100 | F | Asian | Non-hispanic | None | n/a |
| | Moderna-05 | 55.1 | 105 | F | Other | Hispanic | Asthma, migraine, acid reflux | n/a |
| | Moderna-06 | 47.0 | 115 | F | White | Non-hispanic | None | n/a |
| | Moderna-07 | 35.0 | 114 | F | Other | Hispanic | None | n/a |
| | Moderna-08 | 30.7 | 116 | F | White | Non-hispanic | None | n/a |
| | Moderna-09 | 24.6 | 118 | F | White | Non-hispanic | None | n/a |
| | Moderna-10 | 38.1 | 120 | F | White | Non-hispanic | None | n/a |
| | Moderna-11 | 33.4 | 127 | F | Asian | Non-hispanic | None | n/a |
| | Moderna-12 | 36.2 | 127 | F | Other | Hispanic | None | n/a |
| | Moderna-13 | 31.7 | 128 | F | Asian | Non-hispanic | Hyperlipidemia | n/a |
| | Moderna-14 | 48.3 | 128 | F | White | Non-hispanic | None | n/a |
| | Moderna-15 | 40.4 | 131 | F | Black | Non-hispanic | None | n/a |
| | Moderna-16 | 24.2 | 125 | F | White | Non-hispanic | ADHD | n/a |

| | | | | | | | |
|---|---|---|---|---|---|---|---|
| Moderna-17 | 39.7 | 122 | F | White | Non-hispanic | None | n/a |
| Moderna-18 | 25.4 | 127 | M | White | Non-hispanic | None | n/a |
| Moderna-19 | 34.2 | 129 | M | Other | Non-hispanic | Hyperlipidemia, hypertension, non hodgkin's lymphoma, diabetes | n/a |
| Moderna-20 | 37.8 | 138 | F | White | Non-hispanic | migraine | n/a |
| Moderna-21 | 30.4 | 133 | F | White | Non-hispanic | None | n/a |
| Moderna-22 | 38.7 | 126 | M | White | Non-hispanic | None | n/a |
| Moderna-23 | 30.5 | 142 | F | White | Non-hispanic | None | n/a |
| Moderna-24 | 45.1 | 139 | F | Other | Hispanic | None | n/a |
| Moderna-25 | 34.3 | 140 | F | White | Non-hispanic | None | n/a |
| Moderna-26 | 32.6 | 134 | F | White | Non-hispanic | Asthma | n/a |
| Moderna-27 | 30.9 | 125 | F | White | Non-hispanic | None | n/a |
| Moderna-28 | #REF! | 132 | M | White | Non-hispanic | GERD, hyperlipidemia, hypertension, diabetes | n/a |

**Table S2.** Clinical information of the convalescent participants.

| Group | Participant | Age at visit (years) | Days Post-onset or Days post last vaccination dose | Gender | Race | Ethnicity | Notable Comorbidities | Confirmation of COVID test | Disease Features | Hospitalized? | Pulmonary Imaging Findings at Time of Enrollment | COVID-19 or Other Anti-Infective Treatment Given Prior to Enrollment | Outcome |
|---|---|---|---|---|---|---|---|---|---|---|---|---|---|
| **Experienced** | Experienced-01 | 51.2 | 74 | M | White | Non-hispanic | Well controlled HIV (CD4 count >1000) on Biktarvy, asthma | NAAT (PCR) | Headache, blurry vision, dysosmnia, ageusia, fatigue, non-productive cough, dyspnea, hypoxia, diarrhea | Yes | Bilateral infiltrates | Ceftriaxone, Azithromycin | Discharged from hospital |
| | Experienced-02 | 40.4 | 119 | M | White | Non-hispanic | Hyperlipidemia, COVID-19 pneumonia | NAAT (PCR) | Fevers, chills, non-productive cough, dyspnea, hypoxia, headache, diarrhea | Yes | Unilateral infiltrate | Azithromycin, Ceftriaxone | Discharged from hospital |

| | | | | | | | | | | | | | |
|---|---|---|---|---|---|---|---|---|---|---|---|---|---|
| | Experienced-03 | 37.0 | 123 | F | White | Non-hispanic | PCOS | NAAT (PCR) | Non-productive cough, dyspnea, diarrhea, myalgias, muscle spasms, anosmia, ageusia, fatigue, headache | No | Not done | None | Convalescent |
| | Experienced-04 | 46.1 | 103 | M | Other | Non-hispanic | Fatty liver, gastric reflux, vitiligo | NAAT (PCR) | Fever, myalgias, headaches, cough, dyspnea on exertion, fatigue, difficulty concentrating | No | Not done | None | Convalescent |
| | Experienced-05 | 33.7 | 120 | F | White | Non-hispanic | None | Direct lab ELISA anti-S1 IgG | Fever, myalgias, cough, fatigue anosmia/dysgeusia | No | Not done | None | Convalescent |

| | | | | | | | | | | | | | |
|---|---|---|---|---|---|---|---|---|---|---|---|---|---|
| | Experienced-06 | 38.1 | 111 | F | White | Non-hispanic | None | NAAT (PCR) | Fever, cough, body aches | No | Not done | None | Convalescent |
| | Experienced-07 | 59.1 | 86 | F | White | Non-hispanic | None | Direct lab ELISA anti-S1 IgG | | | | | |
| | Experienced-08 | 41.4 | 135 | F | Other | Non-hispanic | None | NAAT (PCR) | 17 days of cough and 7 days of chills, non-productive cough, headache, and nausea | Yes | No infiltrate present | None | Discharged from hospital |
| | Experienced-09 | 40.3 | 126 | M | Asian | Non-hispanic | Fatty liver, gastric reflux, vitiligo | NAAT (PCR) | Fever, myalgias, headaches, cough, dyspnea on exertion, fatigue, difficulty concentrating | No | Not done | None | Convalescent |
| | Experienced-10 | 56.3 | 97 | F | White | Non-hispanic | Enchondroma, hypertension, PONV | NAAT (PCR) | Fever, cough, body aches, nausea, vomiting | No | Not done | None | Convalescent |

| | | | | | | | | | | | | | |
|---|---|---|---|---|---|---|---|---|---|---|---|---|---|
| | Experienced-11 | 54.2 | 124 | F | White | Non-hispanic | Hypertension, history of obesity | NAAT (PCR) | Cough, dyspnea, anosmia | No | Not done | None | Convalescent |
| | Experienced-12 | 43.6 | 76 | M | Other | Hispanic | Diabetes mellitus type 2, hypertension on angiotensin receptor blocker | NAAT (PCR) | Fevers, chills, myalgias, dyspnea, chest tightness, hypoxia | Yes | Bilateral infiltrates | Oseltamivir and Azithromycin prior to admission. Ceftriaxone, Azithromycin, and Lopinavir/Ritonavir during admission. | Discharged from hospital |
| | Experienced-13 | 73.2 | 137 | F | White | Hispanic | On chemotherapy for solid organ malignancy | NAAT (PCR) | Fevers, cough, dyspnea | Yes | Bilateral infiltrates | Vancomycin, piperacillin-tazobactam, azithromycin, ceftriaxone | Discharged from hospital |
| | Experienced-14 | 45.1 | 136 | M | Asian | Non-hispanic | Asthma | NAAT (PCR) | Fatigue, myalgia, productive cough, dyspnea, hypoxia | Yes | Bilateral infiltrates | Ceftriaxone, azithromycin, lopinavir/ritonavir | Required intubation, treated with immunomodulator, improved and was discharged from hospital |

| | | | | | | | | | | | | | |
|---|---|---|---|---|---|---|---|---|---|---|---|---|---|
| | Experienced-15 | 37.0 | 134 | F | White | Non-hispanic | None | None | Headache, myalgias, cough, fevers, chills, dyspnea, non-productive cough, diarrhea, anosmia, ageusia | No | Not done | None | Convalescent |
| | Experienced-16 | 34.3 | 107 | M | White | Non-hispanic | None | NAAT (PCR) | Fever, myalgias, cough, fatigue anosmia/dysgeusia | No | Not done | None | Convalescent |
| | Experienced-17 | 65.2 | 142 | M | White | Non-hispanic | Underlying cardiovascular disease, diabetes | NAAT (PCR) | Cough, low-grade fevers, fatigue, nausea, diarrhea, anosmia | No | Not done | Oseltamivir, unknown antibiotic | Convalescent |
| | Experienced-18 | 39.4 | 129 | F | Asian | Non-hispanic | Anemia | NAAT (PCR) | Fevers, chills, myalgias, rhinorrhea, anosmia, ageusia | No | Not done | None | Convalescent |

| | | | | | | | | | | | | | |
|---|---|---|---|---|---|---|---|---|---|---|---|---|---|
| | Experienced-19 | 40.7 | 120 | M | White | Non-hispanic | Hay fever | NAAT (PCR) | Fever, body aches | No | Not done | None | Convalescent |
| | Experienced-20 | 62.2 | 117 | M | White | Non-hispanic | Hyperlipidemia, Diverticular disease of colon | NAAT (PCR) | Fever, cough | No | Not done | None | Convalescent |
| | Experienced-21 | 31.5 | 126 | M | White | Non-hispanic | None | Direct lab ELISA anti-S1 IgG | Body aches, diarrhea, sore throat | No | Not done | None | Convalescent |

**Table S3.** Antibodies used for flow cytometry experiments.

| Target | Fluorochrome | Clone | Manufacturer | Catalog # |
|---|---|---|---|---|
| **Live/Dead Blue** | - | - | Invitrogen | L23105 |
| **CD3** | APC/Fire 810 | SK7 | Biolegend | 344857 |
| **CD4** | SparkViolet 538 | SK3 | Biolegend | 344674 |
| **CD8** | SparkBlue 550 | SK1 | Biolegend | 344759 |
| **CD14** | BUV805 | M5E2 | BD | 612902 |
| **CD19** | BUV496 | SJ25C1 | BD | 612939 |
| **CD25** | BUV563 | 2A3 | BD | 612919 |
| **CD27** | SB702 | O323 | Invitrogen | 67-0279-42 |
| **CD38** | Qdot655 | HIT2 | Invitrogen | Q22150 |
| **CD45RA** | PE-Cy5.5 | MEM-56 | Life Technologies | MHCD45RA18 |
| **CD56** | BV570 | 5.1H11 | Biolegend | 362539 |
| **CD69** | PE-Dazzle | FN50 | Biolegend | 310942 |
| **CD71** | SB780 | OKT9 | Invitrogen | 78-0719-42 |
| **CD107a** | A647 | H4A3 | Biolegend | 328612 |
| **CD134 (OX40)** | BV421 | ACT35 | Biolegend | 350014 |
| **CD137** | BV750 | 4B4-1 | BD | 747353 |
| **CD154 (CD40L)** | APC-Fire750 | 24-31 | Biolegend | 310848 |
| **CD185 (CXCR5)** | BB515 | RF8B2 | BD | 564624 |
| **CD197 (CCR7)** | BV605 | G043H7 | Biolegend | 353224 |
| **CD200 (OX2)** | PE-Cy7 | OX-104 | Biolegend | 329211 |
| **CD279 (PD-1)** | BB700 | EH12.1 | BD | 566460 |
| **HLA-DR** | BUV661 | G46-6 | BD | 612980 |
| **IgD** | BUV737 | IA6-2 | BD | 612798 |
| **GzmB** | A700 | GB11 | BD | 561016 |
| **IgG** | PerCP-Vio700 | IS11-3B2.2.3 | Miltenyi | 130-119-880 |
| **TNF** | BUV396 | MAb11 | BD | 563996 |
| **IFNg** | BV480 | B27 | BD | 566176 |
| **Perforin** | PE | B-D48 | Biolegend | 353303 |

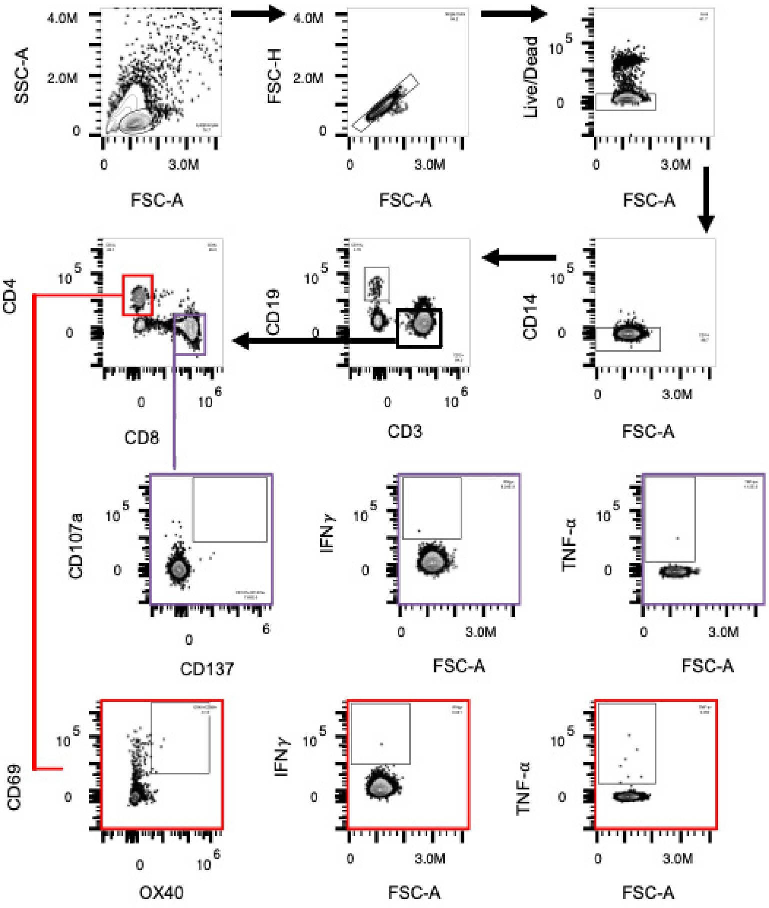


**Figure S1.** CD4+ and CD8+ T cell gating strategy. .The gating scheme for T cell population is shown, with representative flow cytometry plots. FSC (forward scatter) and SSC (side-scatter) channels were used to identify lymphocytes. Purple area indicates CD8+ T cells gating, and red area indicates CD4+ T cells gating.
